# Changes in clinical crown length and the development of gingival recession associated with orthodontic treatment-induced incisor inclination changes: a retrospective cohort study

**DOI:** 10.1093/ejo/cjaf057

**Published:** 2025-07-25

**Authors:** Shatha R Alkhalidy, Budoor S K Bin Bahar, Athanasios E Athanasiou, Miltiadis A Makrygiannakis, M Faysal Talass, Eleftherios G Kaklamanos

**Affiliations:** Private Clinic, P.O. Box: SE45, Abu Dhabi, United Arab Emirates; Al Badaa Health Center, Dubai Health, Al Satwa, Dubai, United Arab Emirates; School of Dentistry, European University Cyprus, 6 Diogenous str., 2404 Nicosia, Cyprus; Hamdan Bin Mohammed College of Dental Medicine, Mohammed Bin Rashid University of Medicine and Health Sciences, Dubai Healthcare City, P.O. Box 505055, Dubai, United Arab Emirates; School of Dentistry, European University Cyprus, 6 Diogenous str., 2404 Nicosia, Cyprus; School of Dentistry, National and Kapodistrian University of Athens, 2 Thivon str., 11527 Athens, Greece; Private Clinic, TECOM, Dubai, United Arab Emirates; School of Dentistry, European University Cyprus, 6 Diogenous str., 2404 Nicosia, Cyprus; Hamdan Bin Mohammed College of Dental Medicine, Mohammed Bin Rashid University of Medicine and Health Sciences, Dubai Healthcare City, P.O. Box 505055, Dubai, United Arab Emirates; School of Dentistry, Aristotle University of Thessaloniki, Aristotle University of Thessaloniki Campus, 54124 Thessaloniki, Greece

**Keywords:** orthodontics, gingival recession, incisor inclination, retrospective study

## Abstract

**Background/Objectives:**

Gingival recession results from the displacement of the gingival margin apically to the cementoenamel junction. There is unclear evidence regarding the impact of orthodontic treatment on the development of gingival recessions. The aim of this study was to investigate the changes in clinical crown length and the development of gingival recession on the labial aspect of the maxillary and mandibular incisors associated with orthodontic treatment and relate these changes to the observed variations in their sagittal inclination.

**Materials/Methods:**

Eighty-two consecutive subjects treated with fixed orthodontic appliances in both dental arches, possessing high-quality pre- and post-treatment dental casts and lateral cephalometric radiographs, were selected from the archives of a private orthodontic clinic. Incisor clinical crown lengths before and after orthodontic treatment, as well as the presence or absence of recession, were measured using digitized study models. Changes in sagittal inclination were assessed from lateral cephalometric radiographs and categorized as proclination, retroclination, or no change (± 1°). Spearman’s correlation coefficient, one-way analysis of variance, and chi-square tests were utilized for analysis.

**Results:**

The mean change in clinical crown lengths for the maxillary incisors ranged from −0.24 to 0.01 mm, while for the mandibular incisors, it varied from 0.06 to 0.10 mm. The inclination changes were −1.78° and 1.03°, respectively, but no correlations were found between these inclination changes and the clinical crown length alterations. Overall, no statistically significant differences were observed in clinical crown length changes concerning the presence of gingival recession among the proclination, retroclination, and no change groups.

**Limitations:**

The sample of this study was retrospective and assessments were carried out immediately post-treatment.

**Conclusions/Implications:**

The alteration of incisor inclination during treatment did not appear to impact the changes in labial clinical crown length and the development of gingival recession in this specific sample.

## Introduction

The harmony of a smile is influenced by various factors, including the shape, position, arrangement, and colour of the teeth, as well as the alignment of the dental and facial midlines [1–3]. In addition to the teeth, the gingival tissues play a crucial role in overall dental aesthetics. The integrity of the mucogingival complex—which comprises tissues that maintain stable and lasting attachments to both the teeth and underlying bone—is fundamental to achieving an aesthetically pleasing outcome [4]. Furthermore, the degree of gingival display [3], along with the relative positioning of gingival margins, significantly influences aesthetic perception [5, 6].

According to the recent classifications of periodontal diseases, gingival recessions are recognized as manifestations of mucogingival deformities [7]. Gingival recession is defined as the apical displacement of the gingival margin, resulting in the exposure of the root surface beyond the cementoenamel junction. This condition can be either localized or generalized in nature [8, 9]. The development of gingival recession is multifactorial and tends to increase in prevalence and severity with age. Primary aetiological factors include oral diseases, aggressive brushing, smoking, poor oral hygiene, and specific anatomical considerations [10, 11].

Recessions most frequently occur on the buccal surfaces of teeth [12], with the mandibular incisors being particularly susceptible to these changes [13]. In addition, premolars are commonly affected in cases where working-side interferences are present during lateral guidance movements [14]. Epidemiological data also suggest a higher prevalence of gingival recession in males compared to females [15, 16]. The clinical consequences of advanced gingival recession include compromised smile aesthetics, dentine hypersensitivity, increased risk of root caries, cervical tooth wear, and greater plaque accumulation [7, 10, 17, 18].

Orthodontic therapy has been associated with alterations in the mucogingival complex, potentially contributing to recession development [19–22]. Although periodontal tissues typically adapt to orthodontic tooth movement [23], this adaptability may be limited in cases involving extreme tooth positions or thin periodontal biotypes [23, 24]. Nevertheless, the current body of evidence remains limited. Even in studies reporting statistically significant differences in recession incidence between proclined and normally inclined incisors, the clinical relevance of such findings is often minimal. Consequently, orthodontic patients with a predisposition to gingival recession should be managed with careful consideration [25]. Moreover, a recent systematic review highlights the shortcomings in current research and suggests that orthodontic patients might encounter an increased risk of developing labial gingival recessions, particularly during the retention phase. However, these conclusions should be interpreted with caution until more robust and high-quality studies are conducted [26].

### Objective

This study aimed to assess changes in clinical crown length and the development of gingival recessions on the labial surfaces of maxillary and mandibular incisors following orthodontic treatment and determine whether these changes are associated with alterations in the sagittal inclination of the teeth.

## Materials and methods

The present retrospective investigation was conducted on orthodontic records (dental study casts and lateral cephalometric radiographs) before (T1) and after the completion (T2) of comprehensive orthodontic treatment. Ethical approval was obtained from the Institutional Review Board of the Mohammed Bin Rashid University of Medicine and Health Sciences, Dubai, UAE (EC1016-004).

### Study sample

We searched all files of patients who had undergone comprehensive orthodontic treatment by means of fixed appliances in both dental arches in the same private orthodontic clinic in Dubai, United Arab Emirates. All patients had been treated with pre-adjusted edgewise fixed orthodontic appliances, 0.022” slot, Roth prescription. The usual sequence of wires was the following: 0.014” NiTi, 0.016” NiTi, 0.016” x 0.022” NiTi, 0.016” x 0.022” SS, 0.016” x 0.022” SS, and 0.017” x 0.025” TMA.

All subjects meeting the following criteria were included in the investigation.

The included patients had to be medically fit Caucasian adolescents or adults, not taking any medication that could affect gingival health (e.g. phenytoin), non-smokers, who had undergone comprehensive orthodontic treatment with fixed appliances in both arches. For these patients to be considered, all their mandibular incisors had to be fully erupted before treatment initiation, and none should have been planned for extraction. Additionally, there should be no generalized visible wear of the incisal edges during treatment. As per the practice treatment protocols, patients were periodically evaluated throughout treatment by a specialist in periodontology. They were considered for the present sample only if they remained periodontally healthy throughout the entire treatment. Regarding the available records, pre- and post-treatment study models with excellent quality of incisor morphology and gingival configuration, along with lateral cephalometric radiographs of similar quality available before treatment (T1) and after treatment (T2), should be accessible. Furthermore, information about treatment duration needed to be provided. Patients who had previously undergone orthodontic treatment or received a combination of orthodontic and orthognathic treatment were not considered for inclusion in this study. Individual teeth with visible wear of the incisal edges that occurred during treatment, or with poor gingival margin anatomy resulting from inaccurate impression technique, were also excluded from further consideration and analysis.

Finally, eighty-two consecutive subjects treated with fixed appliances in both arches, along with high-quality pre- and post-treatment dental casts and lateral cephalograms, were found to meet the inclusion criteria.

### Measurements on digitized dental casts

The dental casts were scanned and digitized by means of the Ortho Insight 3D® Scanner (Motion View Software LLC, Chattanooga, Tennessee, USA). Teeth with visible incisal wear or poor gingival margin anatomy were excluded from further consideration and analysis. The clinical crown length of the assessed maxillary and mandibular incisors was measured blindly by one investigator (BSKBB) on the digitized pre- and post-treatment study models, from the incisal edges of the incisors to the deepest point in the curvature of the gingival margin [27]. The measurements were made using specialized software installed in the Ortho Insight 3D® Scanner. The same investigator also assessed blindly the presence or absence of gingival recession in all maxillary and mandibular incisors on the digitized pre- and post-treatment study models. Labial gingival recession was scored as ‘present’ when the labial CEJ was exposed [27]. From the cases in which all maxillary or mandibular incisors had been assessed, the prevalence rate of gingival recession in at least one maxillary or mandibular incisor was calculated.

The validity of measuring clinical crown lengths and identifying the presence of recession on digital dental casts was investigated in a pilot study. Thirty casts were selected at random. The investigator responsible for the cast measurements assessed blindly the clinical crown length of all maxillary and mandibular incisors on the original plaster casts with an electronic calliper (accurate to 0.0005 inch, approximately 0.01mm) and identified the presence of gingival recession using the aforementioned criteria [27]. The same assessments were performed after the digitization of the stone casts. After one month, all procedures were repeated.

### Measurements on lateral cephalometric radiographs

The pre- and post-treatment lateral cephalometric radiographs were digitized blindly by one investigator (SRA) and used to measure maxillary and mandibular incisor inclination using Viewbox software (dHal, Kifissia, Greece).

The maxillary incisor inclination (U1/NL) was assessed by measuring the angle between the line through the long axis of the maxillary incisor (from incisal edge to incisor apex) and the line connecting the ANS (anterior nasal spine) and PNS (posterior nasal spine) points [28]. Based on the changes observed after orthodontic treatment, subjects were categorized into three groups: a. Retroclined group (ΔU1/NL < 1 °); b. Stable group (ΔU1/NL ± 1 °); and c. Proclined group (ΔU1/NL > 1 °).

The mandibular incisor inclination (L1/MP) was assessed by measuring the angle between the line through the long axis of the mandibular incisor (from incisal edge to incisor apex) and the line along the lower border of the mandible, tangent to the gonion angle and the profile image of the symphysis [29]. Based on the changes observed after orthodontic treatment, subjects were categorized into three groups: a. Retroclined group (ΔL1/MP < 1 °); b. Stable group (ΔL1/MP ± 1 °); and c. Proclined group (ΔL1/MP > 1 °).

### Method error

To determine intra-observer reliability, 30 pre-treatment and post-treatment casts and lateral cephalometric radiographs were randomly selected and blindly measured by the respective investigators and were re-evaluated after one month. To assess potential systematic errors in the measurements of clinical crown lengths and inclination of the incisors, intraclass correlation coefficients and Wilcoxon tests were applied between both sets of measurements. Furthermore, random errors were calculated using the Dahlberg formula [30].


$$\begin{array}{l} s\left(i\right)=\sqrt{\frac{\Sigma \left(Xa-Xb\right)2}{2N}} \end{array}$$

The kappa statistics were used to assess possible errors in the strength of agreement for scoring the presence of recessions between both series of measurements.

### Statistical analysis

Following collection, all data were entered into Microsoft® Excel® sheets (Microsoft® Corporation, USA) and analysed using the SPSS® version 24 software (SPSS® Inc., USA). The assumption of normality was investigated using the Shapiro–Wilk test. To investigate the validity of the digitized measurements, Spearman’s correlation coefficient and the Wilcoxon test were used to analyse the differences between the plaster and digitized cast measurements of incisor clinical crown lengths. The kappa statistic was employed to quantify the agreement between the plaster and digitized cast assessments for the presence of recessions.

The association between the changes in clinical crown length in the labial aspect of the maxillary and mandibular incisors before and after orthodontic treatment with the changes in their sagittal inclination was assessed using Spearman’s rho correlation coefficient. The differences in the changes in clinical crown length between the retroclined, stable, and proclined groups were assessed using the Kruskal-Wallis and Mann-Whitney tests. The association of the changes with age and treatment duration, as well as the differences between genders and Angle Classes, was evaluated using Spearman’s rho correlation coefficient, the Mann-Whitney test, and the Kruskal-Wallis test, respectively.

The differences in the prevalence of gingival recession in the labial aspect of the maxillary and mandibular incisors, before and after orthodontic treatment, among the retroclined, stable, and proclined groups were assessed using Fisher’s exact test. Statistical significance was set at p < 0.05.

## Results

### Validity of digital measurements and method error

The Spearman correlation coefficient for the measurements of incisor clinical crown lengths on the plaster and digitized casts was 0.924. No statistically significant differences were found using the Wilcoxon test. The level of agreement between the plaster and digitized cast assessments for the presence of recessions was very good (kappa scores > 0.800).

Intra-observer repeatability was excellent for both linear and angular measurements. The intraclass correlation coefficients for the measurements of incisor clinical crown lengths and incisor inclination are presented in Table 1.

**Table 1.** Intraclass correlation coefficients [ICC] and 95% Confidence Intervals [95% CI] for clinical crown length and incisor inclination angles.

Maxillary teeth	ICC [95% CI]	Mandibular teeth	ICC [95% CI]
**12 clinical crown length**	0.978 [0.955-0.990]	**32 clinical crown length**	0.950 [0.896-0.976]
**11 clinical crown length**	0.972 [0.941-0.987]	**31 clinical crown length**	0.954 [0.901-0.979]
**21 clinical crown length**	0.983 [0.965-0.992]	**41 clinical crown length**	0.974 [0.946-0.988]
**22 clinical crown length**	0.976 [0.950-0.989]	**42 clinical crown length**	0.980 [0.958-0.991]
** U1/NL angle**	0.982 [0.963-0.991]	**L1/MP angle**	0.964 [0.926- 0.983]

The application of Wilcoxon tests between both series of measurements did not reveal any statistically significant differences. Random errors ranged for the measurements of incisor clinical crown lengths ranged from 0.10 to 0.18 mm, and for incisor inclination ranged from 1.1 to 1.3^o^. The level of agreement between the two series of measurements for the presence of recessions indicated good agreement (kappa scores > 0.800).

### Final sample characteristics

Twelve male and 70 female patients were included in the final sample, with most receiving treatment on a non-extraction basis (76/82). The basic characteristics of the subjects are presented in Table 2. Overall, the maxillary incisors acquired a more palatal inclination, while the mandibular incisors had a more labial inclination after treatment.

**Table 2.** Age characteristics of the sample (years), treatment duration (years), and inclination changes (°).

	Median	Min	Max	Interquartile range
**Age before treatment**	16.12	10.50	40.66	12.50
**Age at the end of treatment**	17.70	10.66	42.33	11.38
**Treatment duration**	1.71	0.08	5.25	1.35
**Change in U1/NL**	-1.2631	-30.64	18.56	9.66
**Change in L1/MP**	0.9698	-20.13	23.31	7.89

### Clinical crown length changes

**Figure 1.**
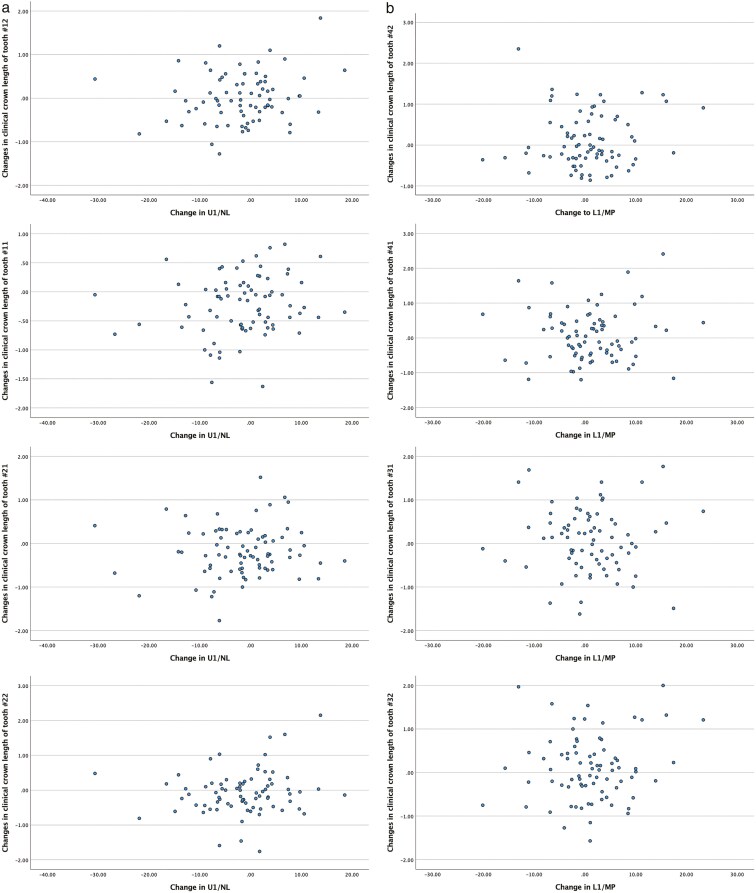
(a to h) Scatterplots illustrating the variations in clinical crown length before and after orthodontic treatment, along with the inclination changes of the maxillary and mandibular incisors.

**Figure 2.**
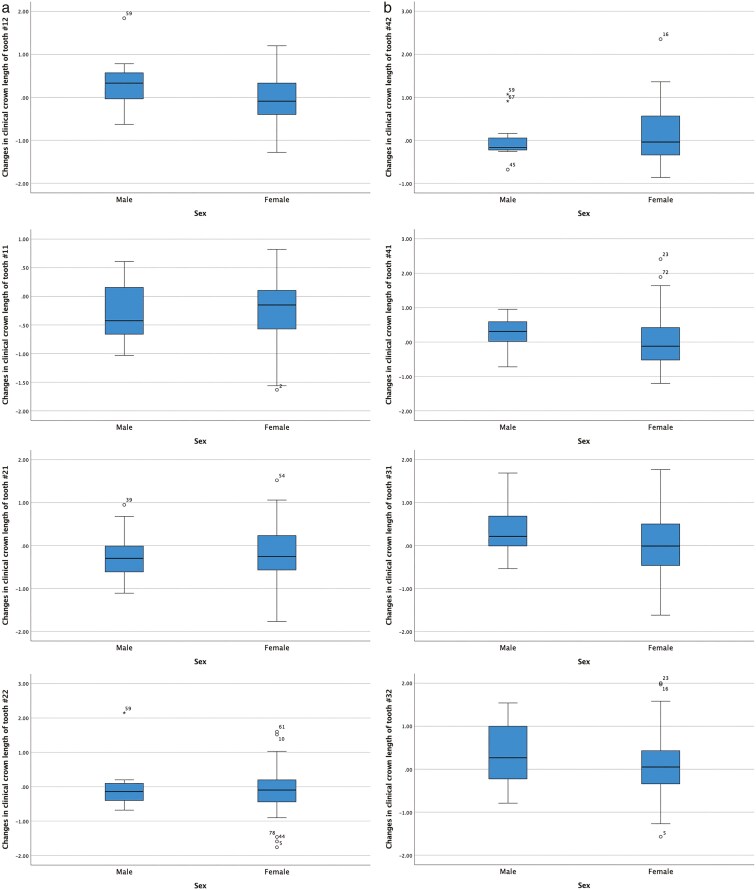
(a to h) Box plots illustrating the variations in clinical crown length of maxillary and mandibular incisors, before and after orthodontic treatment based on sex.

**Figure 3.**
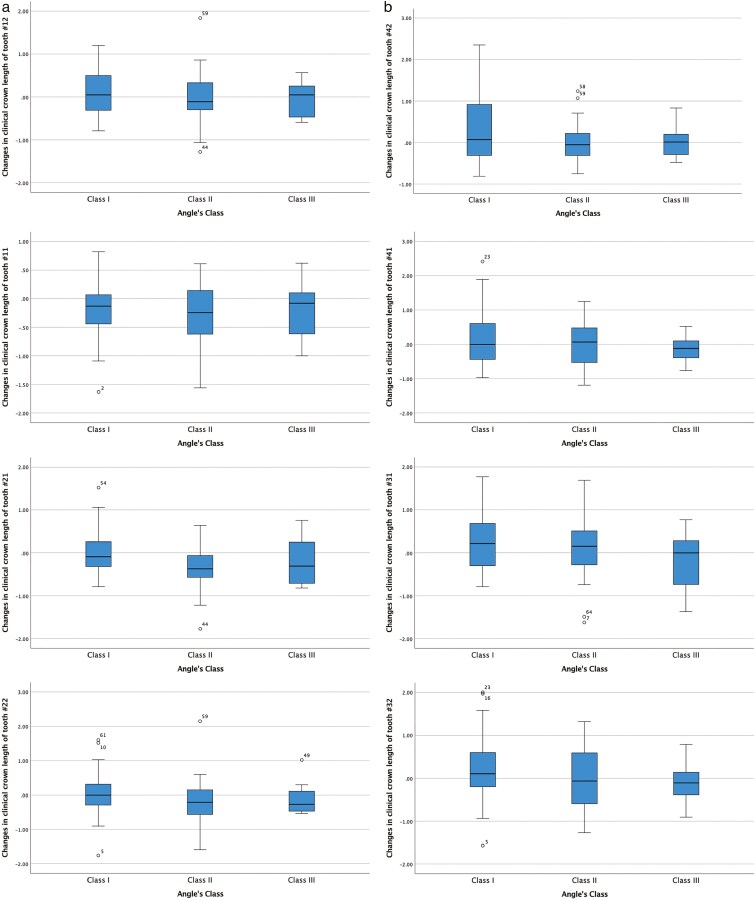
(a to h) Box plots illustrating the variations in clinical crown length of maxillary and mandibular incisors, before and after orthodontic treatment based on Angle’s Class.

**Figure 4.**
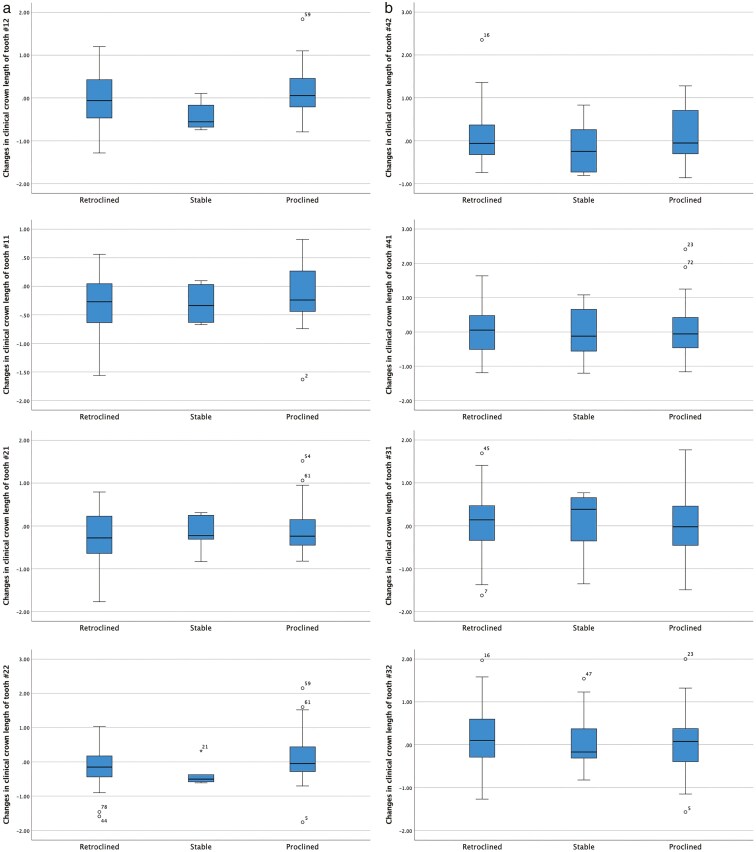
(a to h) Box plots illustrating the variations in clinical crown length of maxillary and mandibular incisors, before and after orthodontic treatment based on retroclined, stable, and proclined groups.

The changes in clinical crown length in the labial aspect of the maxillary and mandibular incisors before and after orthodontic treatment, as well as their correlation with inclination changes of the incisors and treatment duration, are presented in Table 3 and Fig. 1. Overall, clinical crown length decreased in the maxillary teeth while it increased in the mandibular teeth, but no correlation was found with inclination changes or treatment duration. Only the decrease in clinical crown length of tooth 12 was statistically significantly associated with treatment duration. No statistically significant differences in clinical crown length changes were observed between males and females (Table 4 and Fig. 2), among Angle’s Classes (Table 5 and Fig. 3), and retroclined, stable, and proclined groups, except for tooth number 12 (Table 6 and Fig. 4).

**Table 3.** Changes in clinical crown length on the labial aspect of the maxillary and mandibular incisors before and after orthodontic treatment (mm) and their correlation with the inclination changes of the incisors and treatment duration.

	Changes in clinical crown length	Inclination changes	Treatment duration
Tooth	Median; min, max; IR	Spearman’s rho (p value)	
**12**	-0.035; -1.28, 1.84; 0.74	0.144 (0.215)	0.228 (**0.047**)
**11**	-0.240; -1.63, 0.82; 0.72	0.169 (0.136)	0.41 (0.723)
**21**	-0.255; -1.77, 1.52; 0.80	0.064 (0.574)	-0.081 (0.474)
**22**	-0.110; -1.76, 2.15; 0.64	0.118 (0.307)	0.111 (0.335)
**32**	0.070; -1.57, 2.00; 0.79	0.060 (0.602)	0.151 (0.184)
**31**	0.130; -1.62, 1.77; 0.98	-0.057 (0.624)	-0.003 (0.980)
**41**	0.000; -1.20, 2.41; 0.96	-0.001 (0.994)	0.096 (0.399)
**42**	-0.085, -0.86, 2.35; 0.89	0.061 (0.593)	0.180 (0.115)

max: maximum; min: minimum; IR: Interquartile range.

**Table 4.** Differences in clinical crown length changes between males and females (mm).

	Males	Females	
Tooth	Median; min, max; IR	Median; min, max; IR	p-value
**12**	0.395; -0.63, 1.84; 0.70	-0.075; -1.28, 1.20; 0.69	0.053
**11**	-0.310; -1.03, 0.61; 1.06	-0.140; -1.63, 0.82; 0.64	0.552
**21**	-0.130; -0.82, 0.95; 0.68	-0.255; -1.77, 1.52; 0.75	0.647
**22**	-0.060; -0.68, 2.15; 0.58	-0.045; -1.76, 1.60; 0.68	0.890
**42**	-0.180; -0.68, 1.07; 0.59	-0.015; -0.86, 2.35; 1.06	0.221
**41**	0.310; -0.72, 0.95; 0.49	-0.120; -1.20, 2.41; 0.98	0.171
**31**	0.215; -0.54, 1.69; 0.68	0.070; -1.62, 1.77; 1.07	0.115
**32**	0.265; -0.79, 1.32; 1.24	0.065; -1.57, 2.00; 0.92	0.897

max: maximum; min: minimum; IR: Interquartile range.

**Table 5.** Differences in clinical crown length changes among Angle Classes (mm).

	Class I	Class II	Class III	
Tooth	Median; min, max; IR	Median; min, max; IR	Median; min, max; IR	p-value
**12**	0.800; -0.79, 1.20; 0.82	-0.060; -1.28, 1.84; 0.72	0.090; -0.59, 0.57; 0.88	0.743
**11**	-0.140; -1.63, 0.82; 0.51	-0.220; -1.56, 0.61; 0.84	-0.010; -1.00, 0.62; 1.06	0.905
**21**	-0.130; -0.79, 1.52; 0.62	-0.290; -1.77, 0.64; 0.50	-0.230; -0.82, 0.76; 1.22	0.044
**22**	0.025; -1.76, 1.60; 0.63	-0.170; -1.59, 2.15; 0.76	-0.170; -0.54, 1.02; 0.95	0.355
**42**	0.075; -0.81, 2.35; 1.29	-0.040; -0.68, 1.24; 0.52	0.015; -0.48, 0.83; 0.70	0.575
**41**	0.095; -0.96, 2.41; 1.07	0.260; -1.16, 1.25; 1.11	-0.180; -0.76, 0.52; 0.64	0.459
**31**	0.220; -0.79, 1.77; 1.05	0.230; -1.62, 1.69; 1.06	-0.235; -1.37, 0.77; 1.50	0.664
**32**	0.095; -1.57, 2.00; 0.90	0.230; -1.27, 1.32; 1.29	-0.025; -0.91, 0.79; 1.03	0.555

max: maximum; min: minimum; IR: Interquartile range.

**Table 6.** Differences in clinical crown length changes among retroclined, stable, and proclined groups.

	Retroclined	Stable	Proclined	
Tooth	Median; min, max; IR	Median; min, max; IR	Median; min, max; IR	p-value
**12**	-0.06; -1.28, 1.20; 0.99	-0.53; -0.68, 0.11; 0.60	0.055; -0.79, 1.84; 0.68	**0.042**
**11**	-0.13; -1.56, 056; 0.70	-0.52; -0.67, 0.03; 0.59	-0.255; -1.63, 0.82; 0.71	0.298
**21**	-0.25; -1.77, 0.79; 0.87	-0.28; -0.83, 0.31; 0.64	-0.260; -0.82, 1.52; 0.65	0.532
**22**	-0.07; -1.59, 1.03; 0.60	-0.50; -0.61, 0.32; 0.57	-0.045; -1.76, 2.15; 0.78	0.170
**42**	-0.13; -0.74, 2.35; 0.74	0.03; -0.81, 0.83; 1.18	-0.08; -0.86, 1.28; 1.06	0.855
**41**	0.13; -1.19, 1.64; 1.07	-0.18; -1.20, 1.08; 1.29	0.055; -1.16, 2.41; 0.91	0.760
**31**	0.185; -1.62; 1.69; 0.90	0.385; -1.35, 0.77; 1.13	-0.01; -1.49, 1.77; 1.09	0.973
**32**	0.085; -1.27, 1.97; 0.89	-0.02; -0.82, 1.54; 1.63	0.09; -1.57, 2.00; 0.94	0.451

max: maximum; min: minimum; IR: Interquartile range.

The application of paired Mann-Whitney tests for the clinical crown length changes of tooth 12 among the retroclined, stable, and proclined groups revealed a statistically significant difference between the latter two (p = 0.013).

### Gingival recession

**Figure 5.**
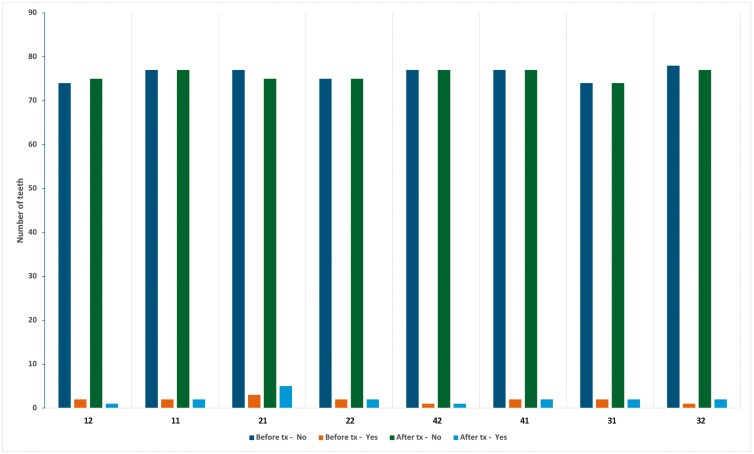
Bar chart illustrating the gingival recession sites before and after orthodontic treatment.

Only a few sites of gingival recession were observed, both before and after treatment (Table 7 and Fig. 5). The differences in the presence of recession between the different teeth, as well as before and after treatment, were not statistically significant (p > 0.05). Similarly, no statistically significant differences in recession presence were noted among the retroclined, stable, and proclined groups. The prevalence of gingival recession in at least one maxillary or mandibular incisor before treatment was 3.7% (3 out of 72) and 1.2% (1 out of 72), respectively. After treatment, the prevalence of gingival recession in at least one maxillary or mandibular incisor was 7.3% (6 out of 72) and 2.4% (2 out of 72), respectively. The difference was not statistically significant (p > 0.05). Likewise, no statistically significant differences were observed between the retroclined, stable, and proclined groups (p > 0.05).

**Table 7.** Changes in the presence of gingival recession on the labial aspect of the maxillary and mandibular incisors before and after orthodontic treatment.

	Gingival recession			
	Before treatment		After treatment	
Teeth	Absence	Presence	Absence	Presence
**12**	74	2	75	1
**11**	77	2	77	2
**21**	77	3	75	5
**22**	75	2	75	2
**42**	77	1	77	1
**41**	77	2	77	2
**31**	74	2	74	2
**32**	78	1	77	2

## Discussion

In general, teeth affected by gingival recession demonstrate an increase in their clinical crown lengths as a result of the accompanying decrease in the vertical height of the attached gingiva [9]. Nevertheless, clinical crown length does not always reliably reflect gingival recession, particularly in cases in which teeth have undergone extrusion without any damage caused to the gingiva, resulting in only a slight increase in crown height, or in instances where tooth wear has caused a decrease in crown length [31].

Gingival recessions are one of the factors that may compromise the final orthodontic therapeutic outcome. During orthodontic treatment, it is possible for teeth to acquire unfavourable positions, leading to apical soft tissue relocation, which results in an increase in clinical crown length and, eventually, the development of recessions [9, 19–22].

Overall, the findings of the present study suggest that changes in incisor inclination during treatment do not affect labial clinical crown length or the development of gingival recession. These findings align with those of a systematic review, which concluded that there is no strong evidence indicating that incisor inclinations can affect periodontal health [32]. Additionally, Allais and Melsen (2003) demonstrated that controlled proclination, when accompanied by good oral hygiene, can be performed in most patients without posing a risk to the periodontium [33]. However, it should be emphasized that new recessions developed in 10% of the investigated teeth, while there was also improvement in 5%. In 85% of their study sample, there was no change. Furthermore, a study by Kamak et al. (2015) showed that neither changes in the inclination of mandibular incisors nor the maintenance of their original positions throughout orthodontic treatment had any impact on the development of gingival recessions in the mandibular incisor region [34]. Although they detected an increase in the clinical crown height of tooth number #32, this alteration was limited to a single tooth, and the changes in clinical crown heights of the remaining incisors were comparable.

However, other studies identified an association between changes in the inclination of lower incisors and an increased risk of gingival recession [24, 35–37]. Artun and Krogstad (1987) studied 40 adult individuals who underwent surgery for mandibular prognathism [24]. Their patients were older at the start of treatment (18−33 years). This may constitute an important factor because the ability of the periodontium to withstand orthodontic treatment appears to decrease with age [38]. They reported that excessively proclined incisors (more than 10˚ to the mandibular plane) showed a significant increase in clinical crown height, causing gingival recession both during appliance therapy and throughout the 3 years of the retention period, compared with patients with minimal change in incisor inclination. However, the long-term prognosis for such teeth with extensive gingival recessions may not be critical. The noted discrepancies may also stem from the fact that IMPA was the only variable measured in lateral cephalograms without accounting for whether the incisors were protracted or retracted. Additionally, it might be due to the more complex aetiology of gingival recession, in which orthodontic treatment is only one factor among others in its development [39].

According to Renkema et al. (2013), only 6.6% of patients were reported to exhibit gingival recessions [27]. Other studies have noted a higher prevalence of patients with gingival recessions following orthodontic treatment [16] (22.9%). This could be attributed to the fact that the authors of the latter publication evaluated recession in patients aged 18−22 years, a sample older than the average age of our own sample. In fact, in the present study, the prevalence of recession after orthodontic treatment was similar to, or slightly smaller than that of untreated patients. Meanwhile, other studies noted lower prevalence rates among patients. Ainamo et al. (1986) reported that 8.7% of teeth among untreated 17-year-olds showed recessions [40], whereas Susin et al. (2004) noted recessions on only 2.9% of teeth among untreated 14−19-year-olds [15]. Finally, a recent study investigated recessions in orthodontically treated patients at the beginning (T1), the end of treatment (T2), and 5 years post-retention. In the treated group, recession incidence was 11.24% at T2 and 67.42% at T3 [41].

In the present study, clinical crown length was measured to provide a secondary indirect measure of gingival recession. Despite the disparities observed in the extent and direction of lower incisor inclination throughout treatment, the increase in clinical crown heights was similar across the various groups, which is a common finding in a significant number of previous investigations [34, 36, 38, 42–47].

An interesting outcome, however not assessed in the present study, was the status of gingival recessions after the retention period. According to Celis et al. (2025), they usually show a surge. This result cannot be corroborated in the present study since the observation period ends after treatment. Those findings suggest that while orthodontic treatment and retention are essential for the maintenance of the orthodontic result, they may contribute to gingival recession, underlining the need for careful periodontal monitoring during and after treatment [41].

The width of the zone of keratinized gingivae is another parameter that has also been investigated in the literature. Various studies have explored this matter. One of the early studies on this topic, conducted by Bowers (1963), found that lingually displaced teeth were those that often presented the greatest width of keratinized gingiva [48]. It appears that when these teeth were brought into proper alignment via orthodontic therapy, the result was a decrease in their width. Similarly, in the late 70s, Dorfman (1978) concluded that lingual tooth movement results in an increased bucco-lingual thickness of the tissue at the facial aspect of the tooth, which leads to coronal migration of the soft tissue margin (decreased clinical crown height) [49]. Conversely, facial tooth movement resulted in reduced bucco-lingual tissue thickness, thereby lowering the height of the free gingival portion and increasing clinical crown height. A few years later, in 1981, a retrospective study by Coatoam et al. assessed the effect of orthodontic therapy on the width of the zone of the keratinized gingivae and showed that statistically significant increases in the clinical crown during orthodontic therapy did not correspond to statistically significant decreases in the width of keratinized gingivae [50]. Even minimal widths of keratinized gingiva (less than 2 mm) were capable of withstanding the stresses exerted by orthodontic mechanics. This specific change is partly related to the position of the tooth in the dental arch and the pre-treatment condition of the keratinized gingiva. The greatest loss of width of keratinized gingiva occurred in the area of the lateral incisors. Perhaps this resulted from the fact that both maxillary and mandibular lateral incisors in this investigation were often displaced lingually. Moreover, a recent publication detected a significant difference in the width of keratinized gingivae between patients who had undergone orthodontic treatment with and without extractions [46]. Nevertheless, a very recent study by Kloukos et al. (2025) suggested that lower incisor proclination during orthodontic treatment does not significantly alter gingival thickness and width of keratinized gingivae, but orthodontic treatment, overall, does result in a reduction of the width of keratinized gingivae [51].

Regarding other potential contributing factors to the development of recessions, Lee and co-workers (2020) demonstrated that initial gingival and bone thickness, tooth positioning, and rotation do not appear to significantly influence the occurrence of gingival recessions [52]. Additionally, Kalina et al. (2021) suggested that orthodontic treatment in adults does not necessarily increase the risk of labial gingival recession, provided that the patient’s periodontal biotype is taken into account and the roots remain within the limits of the alveolar bone [53]. Nevertheless, other anatomical and treatment-related elements—such as an increased symphysis height, a higher symphysis height-to-crestal width ratio, and even substantial changes in the inclination of the lower incisors—have been linked to a greater likelihood of recession development [54].

In the present study, the assessment of recessions was performed on digital casts, whereas a previous similar investigation by Renkema et al. [27] was conducted on plaster casts. Other techniques for evaluating and quantifying gingival recessions include direct probe measurements, digital photography measurements, as well as 3D superimposition and non-superimposition methods [9]. All these methods have various potential sources of error. For example, the angulation of the probe during measurement or the shooting angle in photographs may affect the quantification of gingival recession.

Overall, the level of available evidence is low, and the degree of recession found in studies with statistically significant differences between proclined and normally inclined teeth is of questionable clinical importance; it should be treated with caution until additional research becomes available [25].

### Limitations

Although we followed a similar methodology to other investigations [33, 42, 43], the fact that assessments were carried out immediately post-treatment may, at some point, limit the applicability and generalizability of the observed results. It is possible that recessions could become clinically detectable over a longer period. In fact, Artun and Krogstad (1987) suggested an observation time of 3 years; during this period, the clinical crown height increased significantly more in patients with excessive proclination than in those with minimal change in incisor inclination [24]. However, thereafter, the differences between the groups were not significant. Additionally, a very recent study by Theodorelos et al. (2024) recruited a sample of patients with a mean observation period of 15.7 years since the end of their treatment [55]. Renkema et al. (2015) also found no difference in recession 5 years after treatment between individuals with an average final IMPA of 90.80 and those who finished treatment at 105.2˚ [47]. Immediately after treatment, gingival inflammation and enlargement may mask recession. In the present study, despite the immediate post-treatment evaluation of recessions, it should be noted that high-quality clinical appearances of thin gingival margins and pointed papilla were present. Finally, it should be noted that at each individual tooth site, the number of discordant pairs—cases where a change in recession presence was noted—was minimal. The observed differences, which were not statistically significant, may stem from the small extent of clinical changes in either direction. For instance, certain teeth (e.g. #12) displayed a slight reduction in gingival recession, while others (e.g. #21) experienced a minor increase. These differences were subtle and not consistently directional, suggesting overall stability in gingival recession following treatment. However, we recognize that the possibility of Type II error cannot be entirely dismissed, and some subtle clinical changes may have gone undetected.

Another factor that may limit the generalizability of the findings of this study is that all orthodontic treatments were conducted in a single private orthodontic clinic. Additionally, it is important to note that all patients were Caucasian. According to the existing literature, Asian or Asian-American individuals tend to exhibit a thinner gingival phenotype compared to African-American or Caucasian individuals. In fact, Hsu et al. (2020) reported that Asian-Americans more often display a thin tissue phenotype, diminished keratinized gingiva, and greater gingival recession, whereas African-Americans show a thicker phenotype and larger tooth/root dimensions [56]. Similarly, Kim et al. (2020) observed that Asian individuals typically have thinner gingiva than Caucasians, a characteristic associated with a higher prevalence of recession [57]. In contrast, Zweers et al. (2014) did not report ethnic differences when classifying periodontal biotypes into thin scalloped, thick flat, and thick scalloped categories [58]. These parameters may limit the generalizability of our findings to other populations or clinical contexts.

The inability to assess a potential impact on the width of the attached gingiva in the observed results represents another limitation of the present study. From a practical perspective, this is a clinical parameter that cannot be measured from study casts [43]. Moreover, both the extent of gingival recession and the inclination of the lower incisors were measured on pre-treatment and end-of-treatment records, but not throughout the treatment. It is possible that in some subjects, the lower incisors may have experienced round-tripping: they might have initially been moved labially to correct a discrepancy, which may have led to recession, and subsequently, been moved lingually (retroclined) before the final records were taken. In such cases, the transient recession would go undetected by relying solely on the pre- and post-treatment records. Lastly, details about smoking and oral hygiene were not recorded in the retrieved documents. Therefore, the influence of these variables could not be assessed and incorporated into the correlation analysis.

Another significant limitation is related to the use of lateral cephalograms. This methodological issue is prevalent in most existing literature. One of the main problems in correlating incisor proclination with gingival recession and identifying patients prone to this side effect may be associated with the amount of bone supporting the tooth roots in the buccolingual dimension. The drawback of 2D imaging is that it does not provide any information regarding the hard tissue biotype. It is well documented that bone thickness in the anterior maxillary and mandibular areas can range from very thin to absent, indicating that dehiscences and fenestrations may already be present before the initiation of orthodontic treatment. Additionally, cortical bone thickness can vary according to the patients’ skeletal patterns. A study using CBCTs as an assessment method to correlate bone thickness, incisor inclination, and the development of gingival recessions could benefit diagnosis and potentially establish cut-off values for both parameters, aiding the prediction of susceptibility and risk of future gingival recession development after the completion of treatment.

A very recent systematic review contends that the presence of a thin gingival phenotype, the pre-treatment existence of gingival recession, the baseline width of keratinized gingiva, and facial gingival margin thickness may be correlated with an increased risk of gingival recession development [59]. As mentioned above, these factors were not considered in the present study.

Due to the limitations of the existing investigations, further studies are needed to better understand the extent of the influence arising from each clinical variable [60].

### Recommendations for future research

Further well-designed, prospective longitudinal clinical studies, including assessments of oral hygiene and gingival condition before, during, and after treatment, are needed to clarify the relationship between orthodontic treatment and gingival recession, particularly regarding changes in incisor inclination. Short-term investigations, over a 1–2 year period, that integrate both orthodontic and periodontal parameters may offer clearer insights, as long-term observations often blur the distinction between recession caused by tooth movement and that resulting from plaque-related periodontal disease. To enhance understanding, future research should also consider additional periodontal and mucogingival factors that influence gingival recession, as well as patient-related variables such as oral hygiene practices and lifestyle habits that impact periodontal health.

## Conclusion

In this retrospective sample of primarily non-extraction cases, changes in incisor inclination during treatment did not seem to increase labial clinical crown length or contribute to gingival recession immediately after treatment. These findings mainly pertain to cases involving routine changes in incisor inclinations and should not be generalized to treatment plans involving extreme proclination or retroclination. Since gingival recessions may develop or progress during the retention period, further longitudinal follow-up is necessary to fully evaluate the long-term periodontal impact of orthodontic tooth movement.

## Data Availability

The datasets generated and/or analysed during the current study are available from the corresponding author on reasonable request.
